# Current Socioeconomic Status Correlates With Brain Volumes in Healthy Children and Adolescents but Not in Children With Prenatal Alcohol Exposure

**DOI:** 10.3389/fnhum.2020.00223

**Published:** 2020-06-30

**Authors:** Kaitlyn McLachlan, Dongming Zhou, Graham Little, Carmen Rasmussen, Jacqueline Pei, Gail Andrew, James N. Reynolds, Christian Beaulieu

**Affiliations:** ^1^Department of Psychology, College of Social & Applied Human Sciences, University of Guelph, Guelph, ON, Canada; ^2^Department of Zoology, Kunming Medical University, Kunming, Yunnan, China; ^3^Department of Biomedical Engineering, Faculty of Medicine and Dentistry, University of Alberta, Edmonton, AB, Canada; ^4^Department of Pediatrics, Faculty of Medicine and Dentistry, University of Alberta, Edmonton, AB, Canada; ^5^Department of Educational Psychology, Faculty of Education, University of Alberta, Edmonton, AB, Canada; ^6^Glenrose Rehabilitation Hospital PAE Clinic, Edmonton, AB, Canada; ^7^Department of Biomedical and Molecular Sciences, School of Medicine, Faculty of Health Sciences, Queens University, Kingston, ON, Canada

**Keywords:** brain volume, development, fetal alcohol spectrum disorder (FASD), prenatal alcohol exposure (PAE), socioeconomic status (SES)

## Abstract

Individuals with prenatal alcohol exposure (PAE) exhibit neurological deficits associated with brain injury including smaller brain volumes. Additional risk factors such as lower socioeconomic status (SES) may also have an impact on brain development for this population. This study examined how brain volumes are related to SES in both neurotypically developing children and adolescents, and those with PAE. 3D T1-weighted MPRAGE images were acquired from 69 participants with PAE (13.0 ± 3.2 years, range 7.1–18.8 years, 49% female) and 70 neurotypical controls (12.4 ± 2.9 years, range 7.0–18.5 years, 60% female) from four scanning sites in Canada. SES scores calculated using Hollingshead’s Four-Factor Index of Social Status from current caregiver placement were not significantly different between groups, though more children with PAE had lower SES scores compared to controls. Psychometric data comprised 14 cognitive measures, including executive functioning, attention and working memory, memory, math/numerical ability, and word reading. All cognitive scores were significantly worse in children with PAE compared to controls, though SES was not correlated with cognitive scores in either group after correction for multiple comparisons. All 13 brain volumes were smaller in children with PAE compared to children in the control group. Higher SES was associated with larger hippocampus and amygdala volumes in controls, but there were no such associations in children with PAE. Direct evaluation of the interaction between SES and diagnostic group did not show a significant differential impact of SES on these structures. These findings support previous links between SES and brain volumes in neurotypically developing children, but the lack of such a relationship with SES in children with PAE may be due to the markedly smaller brain volumes resulting from the initial brain injury and postpartum brain development, regardless of later SES.

## Introduction

Prenatal alcohol exposure (PAE) is associated with a range of lifelong physical, cognitive, and neurological impacts, and may result in a diagnosis of fetal alcohol spectrum disorder (FASD; Cook et al., 2016; Hoyme et al., 2016; Mattson et al., 2019). PAE can impact any stage of fetal development, with studies suggesting that structural impacts are associated with the developmental timing of exposure (see reviews by Jones, 2011; Mattson et al., 2019). For instance, alcohol can impact gastrulation leading to facial dysmorphology as early as weeks 3 and 4 of pregnancy (Sulik, 2005). Alcohol also crosses the placenta and blood-brain barrier causing decreased protein synthesis, reduced DNA translation, and irreversible brain injury such as neuronal death, among other effects (West et al., 1994; Miller, 1996). PAE can also trigger a range of additional impacts such as maternal hypoxia, oxidative stress, displacement or malabsorption of essential nutrients, and altered metabolism, all of which can further alter fetal brain development (for reviews see Goodlett and Horn, 2001; Young et al., 2014; del Campo and Jones, 2017). Smaller brain volumes are commonly seen in children with PAE, as identified in autopsy (Jones and Smith, 1975; Clarren and Smith, 1978) and by several *in vivo* quantitative neuroimaging techniques (for reviews see Norman et al., 2009; Lebel et al., 2011). General population prevalence for FASD ranges from 2 to 5%, however, the disability is thought to occur more frequently in communities marked by socioeconomic disadvantage (May et al., 2014, 2018; Lange et al., 2017; Popova et al., 2019a,b).

Socioeconomic status (SES) has an impact on brain development and cognitive function in typically developing children (for reviews see Bradley and Corwyn, 2002; Hackman and Farah, 2009; Hackman et al., 2010; Brito and Noble, 2014). Poverty likely shapes brain development through a series of complex factors, including maternal deprivation, environmental stressors, and environmental toxins, among others. Together these factors may lead to neural changes *via* biological mechanisms including gene × environment interactions, epigenetic modifications, and hypothalamic pituitary adrenal (HPA) function. Ultimately this has been associated with key structural and functional changes in the brain, as well as observed neurocognitive and academic outcomes (see reviews by Brito and Noble, 2014; Johnson et al., 2016). In typically developing children, lower SES is usually associated with worse cognitive performance and academic achievement (Willms, 2004; Farah et al., 2006; Johnson et al., 2016), increased health concerns (Chen et al., 2002; Adler and Ostrove, 2006), and smaller brain volumes (Jednoróg et al., 2012; Noble et al., 2012; Cavanagh et al., 2013). Also, women and families experiencing poverty and lower SES may be at increased risk for poor maternal nutrition during pregnancy and increased maternal stress, in addition to alcohol and other substance use in the context of limited social determinants of health (Bradley and Corwyn, 2002; Lewis et al., 2011; von dem Knesebeck et al., 2013; Young et al., 2014). Lower SES has also been linked to additional mental health disorders, with generally higher levels of depression, anxiety, and psychosis (Lorant et al., 2003; Kessler et al., 2005; McLaughlin et al., 2012; Agerbo et al., 2015; Blair and Raver, 2016; Vukojević et al., 2018). Further, some studies explicitly link SES with reduced regional brain volumes, such as in adults with schizophrenia relative to controls (Takayanagi et al., 2010; Yeo et al., 2014).

Given the high rates of additional pre and postnatal experiences of adversity reported in children with FASD, coupled with higher rates of FASD in the context of health and social inequities, SES may be an important contributing factor for alterations in brain development for children and adolescents with PAE (Streissguth et al., 2004; May et al., 2005, 2008; McLachlan et al., 2015; Lebel et al., 2019; Popova et al., 2019b). While FASD can occur following an alcohol-exposed pregnancy for women and families of any SES level, lower SES is an important factor that elevates the risk of having a child with FASD, and as noted, several epidemiological studies have shown lower SES for families of children with FASD (e.g., see May et al., 2011; Popova et al., 2019b). Recently, Uban et al. (2020) reported findings from a study of SES and brain structure in a U.S. cohort of 95 children with PAE and 102 age and sex-matched controls. Their results indicated that higher SES in the current child placement was associated with larger subcortical volumes in neurotypically developing children, but not in children with PAE. To our knowledge, no other studies have evaluated SES-brain structure relationships in children with PAE or FASD. Thus, the present study aimed to identify whether current SES is associated with cognitive functioning and brain volume in children and adolescents with PAE compared to neurotypical controls.

## Materials and Methods

### Participants

Participants for the current study were 139 children and adolescents drawn from the larger NeuroDevNet FASD cohort (*N* = 239, Reynolds et al., 2011), based on their status for MRI, age, and other exclusion criteria (as outlined below). Children with PAE (*n* = 69) were recruited through FASD diagnostic clinics at six Canadian sites (although MRI was only at 4 sites), including Kingston, ON; Ottawa, ON; Edmonton, AB; Cold Lake, AB; Winnipeg, MB; and Vancouver, BC. Neurotypically developing children (*n* = 70) were recruited as a control sample from the same geographic regions, matched as closely as possible for age and sex, and were excluded if they had any neurological or psychiatric disorders.

Participants in the FASD group were predominantly assessed before their participation *via* a multidisciplinary team that adhered to the 2005 Canadian Diagnostic Guidelines (Chudley et al., 2005). FASD diagnosis was made by experienced multidisciplinary teams using the Canadian Guidelines from 2005 that incorporate objective methods of evaluating core clinical features of FASD, including neurodevelopmental impairment, facial dysmorphology, growth deficiency, PAE, and other pre and post natal adversity factors. As part of the diagnostic process, PAE is evaluated using a range of reliable sources of information. While detailed information regarding the pattern, timing, and volume of exposure was not available for the current study, records were reviewed to ensure that participants with PAE had confirmed exposure at the above risk thresholds following the Washington Diagnostic and Prevention Network FASD 4-digit classification system (Astley, 2004). Most children in the PAE group were diagnosed with FASD under the Canadian Guidelines^1^, including 26% (*n* = 18) with fetal alcohol syndrome (FAS) or partial fetal alcohol syndrome (pFAS), and 51% (*n* = 35) with alcohol-related neurodevelopmental disorder (ARND). An additional 23% (*n* = 16) had confirmed PAE but did not meet the criteria for formal diagnosis or were deferred for re-evaluation.

### Socioeconomic Status

Socioeconomic status (SES) for the current caregiver placement at the time of scanning was calculated using Hollingshead’s Four-Factor Index of Social Status, a commonly used estimate for SES based on the highest educational and current occupation for one or two caregivers in the household (Hollingshead, 1975; Adams and Weakliem, 2011). For the current study, caregivers who attended the study visit provided education and occupation data for one or both contributing adult caregivers/guardians living in the child’s household, and weighted SES scores were derived based on established occupational and educational attainment scores. Occupational scores range from 1 (e.g., service workers) through 9 (e.g., higher executives and major professionals), while educational scores range from 1 (e.g., lower than 7th-grade education) through 7 (e.g., graduate-level education). The Hollingshead Index continues to be among the most widely used, brief, valid, and reliable estimate of SES in health and imaging research (e.g., Bornstein et al., 2003; Lawson et al., 2013, 2017; Cohen-Zimerman et al., 2019; Spann et al., 2019).

### Cognitive Tests

Children completed a single testing session comprising a broad battery of cognitive measures spanning domains including executive functioning, attention and working memory, memory, numerical ability, and word identification (see McLachlan et al., 2017). In the current study, measures were drawn from the NEPSY-II (Korkman et al., 2007), including Animal Sorting (assessing basic concept formulation and set-shifting), Inhibition (including Naming, Inhibition, and Switching, measuring the inhibition of automatic responses in favor of novel responses and switching between response types), Memory for Names (assessing short- and long-term verbal learning and retention), and Auditory Attention and Response Set (assessing selective and sustained attention, and ability to shift and maintain information while inhibiting previously learned responses). NEPSY-II scores are age-normed as scaled scores (*M* = 10, and *SD* = 3) with higher scores indicating better cognitive performance relative to same-aged peers. Participants also completed subtests from the Working Memory Test Battery for Children (WMTB-C, Pickering and Gathercole, 2001), including Digit Recall (verbal/phonological working memory) and Block Recall (visuospatial working memory). WMTB-C age-referenced standardized scores were calculated (*M* = 100, and *SD* = 15). Participants completed the Quantitative Concepts subtest (quantitative reasoning and mathematics knowledge) from the Woodcock Johnson-III Tests of Achievement (WJ-III ACH, Woodcock et al., 2001) and the Word Identification subtest (word identification/reading ability) from the Woodcock Reading Mastery Tests-Revised (WRMT-R, Woodcock, 1998). Both tools use age-referenced standard scores (*M* = 100, and *SD* = 15).

Caregivers provided ratings of each child’s executive functioning in everyday contexts *via* the Behavior Rating Inventory of Executive Function (BRIEF, Gioia et al., 2000). The Behavior Regulation Index (BRI, a summary score tapping one’s ability to shift cognitive set and modulate appropriate behavior through effective behavioral control) and the Metacognition Index (MI, a summary of subscales tapping the ability to cognitively self-manage tasks and monitor performance) were used in analyses for the current study, specifically, age and sex referenced *t*-scores, where higher scores indicate greater levels of difficulty (*M* = 50, *SD* = 10). Caregivers also completed a short interview and provided basic demographic information about their child, including age, sex, handedness, ethnicity, and caregiving placement. Imaging sessions were typically conducted during a second session, within a few days or weeks of the cognitive testing session. Cognitive testing was typically completed before the MRI session.

The Human Research Ethics Boards at Queen’s University, the University of Alberta, the Children’s Hospital of Eastern Ontario, the University of Manitoba, and the University of British Columbia reviewed and approved all study procedures. A parent or legal guardian gave written informed consent, and children provided assent before study participation.

### Image Acquisition and Processing

From the overall NeuroDevNet sample, 177 participants underwent brain MRI including 3D T1-weighted MPRAGE (1 × 1 × 1 mm^3^ in ~5–6 min; for acquisition details see Little and Beaulieu, 2020) at four imaging sites in Canada (University of British Columbia, UBC, 3T Philips Intera; University of Alberta, UofA, 1.5T Siemens Sonata; University of Manitoba, UofM, 3T Siemens Trio; and Queen’s University, 3T Siemens Trio). In total, 20 participants were excluded after visual inspection for motion artifacts and quality control from the CIVET quality control program for segmentation and surface extraction, including eight controls and nine PAE from motion artifacts, and two controls and one PAE from segmentation errors in local areas. Another seven participants were removed owing to exclusions (e.g., neurological disorder for controls, unclear data for PAE) and 11 participants were removed owing to missing SES scores and age <7 years (several cognitive tasks are designed for children ages 7 and above), leaving a final sample of 70 neurotypically developing control children and 69 children with PAE/FASD.

An automated program (Freesurfer 5.1, Fischl et al., 2002) on the CBrain platform^2^ was used to yield 13 volumes of the total cerebrum, total gray matter (GM), cortical GM, and total deep subcortical GM along with its individual subregions including the hippocampus, amygdala, thalamus, caudate, putamen, and pallidum, white matter (WM), and cerebellum GM and WM. Left and right volumes were measured separately and then combined to reduce multiple comparisons.

### Inter-site Correction

Given the potential for scanner differences across the four imaging sites to yield systematically differential quantitative image metrics, including volumes (e.g., vendor, model, field strength, etc., see Han et al., 2006; Wonderlick et al., 2009; Chalavi et al., 2012; Jovicich et al., 2013), volumes for the current study were corrected based on the consistency of these metrics from the same 8 healthy participants each scanned twice at each site, i.e., 64 scans total, as was done previously (Zhou et al., 2018). Effects of site, scan, and site-by-scan interactions were tested using two-way repeated-measures analysis of variance (RM-ANOVA). For a specific volume (e.g., the total cerebrum volume), if the site was significantly biased (*p* < 0.05), the volume was corrected for each site. A correction factor for each site was calculated as a Δ volume from the difference between the volume at each site and the mean volume across all four sites. The corrected volume was then determined for each control and PAE participant per site.

### Statistics

Demographic differences between groups were assessed by *t*-test for continuous variables (age, SES), Chi-square tests for dichotomous variables (e.g., handedness, sex), and non-parametric Wilcoxon Rank Sum tests for categorical variables (current caregiver, ethnicity, scanning site). Group comparisons for cognitive scores were made using *t-*tests, while brain volume comparisons were evaluated using analysis of covariance (ANCOVA), with both age and sex included as covariates. Associations between SES and cognitive scores were evaluated using Pearson correlations within each of the control and PAE groups separately. SES and brain volume associations were assessed using linear regression, controlling for age and sex, in each of the control and PAE groups separately. Exploratory hierarchical linear regressions were also conducted to directly assess the possible differential impact of SES on brain volumes in children with PAE compared to controls. Covariates (age, sex) were included in the first step of each model, followed by both group and SES (mean-centered) in the second step, and the interaction between the group and SES (mean-centered) in the third step. In all inferential analyses, *p*-values were considered significant at a level of 0.05. Multiple comparisons were corrected in primary analyses (e.g., between-group comparisons, bivariate associations) using the Benjamini–Hochberg false discovery rate (FDR; *α* = 0.05, *q* = 0.10 for each family of comparisons; Benjamini and Hochberg, 1995, 2000). Effect sizes for *t-*tests (Cohen’s *d*), chi-square (phi, *φ*), and *F* tests (partial eta squared, $\eta_{\text{p}}^{2}$) are reported. Cohen’s *d* values range from 0.2 (small) to 0.5 (medium) to 0.8 and above (large), *φ* values range from 0.1 (small) to 0.3 (medium) to 0.5 and above (large), and $\eta_{\text{p}}^{2}$ values range from 0.02 (small), to 0.13 (medium) to 0.26 and above (large; Cohen, 1988). Analyses were conducted using IBM SPSS Statistics 26 for Mac.

## Results

### Demographics

**Figure 1:**
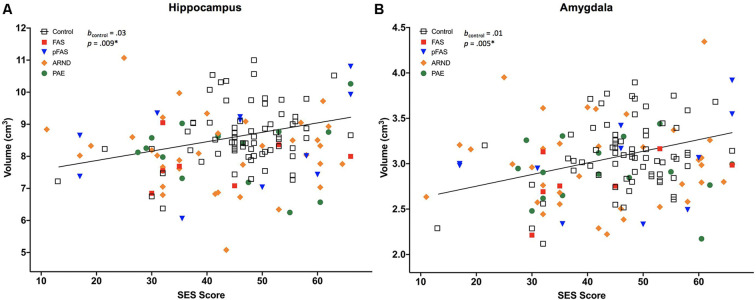
Two of 13 brain volumes, **(A)** hippocampus, and **(B)** amygdala, were positively associated with socioeconomic status (SES) in neurotypically developing controls (*n* = 70), but not in prenatal alcohol exposure (PAE), *n* = 69 [displayed for different PAE/fetal alcohol spectrum disorder (FASD) subgroups, including fetal alcohol syndrome (FAS), *n* = 7; partial FAS (pFAS), *n* = 11; alcohol-related neurodevelopmental disorder (ARND), *n* = 35; and PAE, *n* = 16]. The *indicates that the *p*-value remained significant after correction for multiple comparisons, see Table 4. Regression lines pictured do not include covariates.

Age, sex, and handedness were similar between typically developing control participants (12.4 ± 2.9 years, range 7.0–18.5 years, 60% female, 93% right-handed) and participants with PAE (13.0 ± 3.2 years, range 7.1–18.8 years, 49% female, 91% right-handed, see Table 1). While mean SES did not differ significantly between groups, who shared comparable ranges (47 ± 9, range 13–66 in controls and 43 ± 14, range 11–66 in PAE, *p* = 0.06), the distributions for each group differed considerably, with only 11/70 controls (16%) having relatively lower SES scores (e.g., <40) as compared to nearly three times that number for children with PAE (30/69, 44%; see Figure 1). SES did not differ significantly across PAE subgroups, though the SES range was somewhat restricted in the PAE-only subgroup (44 ± 13, range 28–66) compared to the FAS/pFAS (44 ± 16, range 17–66) and ARND subgroups (42 ± 14, range 11–65). Differences in SES were apparent by site, *F*_(3,135)_ = 2.92, *p* = 0.036, $\eta_{\text{p}}^{2}$ = 0.06). Mean SES scores were highest at Queen’s site (PAE 52 ± 11, range 32–66, Control 45 ± 5, range 32–51), followed by the UofM site (PAE 45 ± 20, range 17–66, Control 50 ± 7, range 37–58), and then the UBC (PAE 36 ± 17, range 17–66, Control 47 ± 12, range 22–66) and UofA sites (PAE 38 ± 11, range 11–61, Control 47 ± 9, range 13–63). Current caregiving arrangement differed significantly between groups, with nearly all children in the control group (*n* = 69, 99%) living with their biological parent(s), compared to only 9% (*n* = 6) of those with PAE, and the rest residing in adoptive families (*n* = 42, 61%), foster care placements (*n* = 12, 17%), or with other legal guardians (*n* = 7, 13%). More participants were recruited and scanned in Alberta compared to other sites (e.g., 35 controls, 50%, and 30 PAE, 43%), but there was roughly an equal number of participants in each group at each site.

**Table 1:** Demographic information for control and prenatal alcohol exposure (PAE) groups (*N* = 139).

			Comparison^a^		Subtypes		
Controls	PAE	*p*	*d* (*φ*)	FAS/pFAS	ARND	PAE
*n*	70	69			18	35	16
Age *M* ± SD (range)	12.4 ± 2.9 (7.0–18.5)	13.0 ± 3.2 (7.1–18.8)	0.223	0.21	12.4 ± 3.1 (7.8–18.5)	13.5 ± 3.2 (7.7–18.8)	12.5 ± 3.1 (7.1–17.7)
SES *M* ± SD (range)	46.8 ± 8.9 (13–66)	42.9 ± 14.2 (11–66)	0.059^c^	0.19	43.6 ± 15.9 17–66	42.3 ± 14.3 11–64.5	43.5 ± 12.7 27.5–66
Sex % female	60.0	49.3	0.20	−0.11	55.6	51.4	37.5
Handedness % right^b^	92.5	90.8	0.71	−0.03	88.2	94.1	85.7
Current caregiver %								
Biological parent	98.6	8.7	<0.001	0.90	5.6	11.4	6.3
Adoptive family	0.0	60.9			66.7	62.9	50.0
Foster care	0.0	17.4			16.7	20.0	12.5
Other guardian	1.4	13.0			11.1	5.7	31.3
Ethnicity %								
Caucasian	90.0	33.3	<0.001	0.60	50.0	31.4	18.8
Indigenous	1.4	43.5			33.3	40.0	62.5
Other^c^	8.6	23.2			16.7	28.6	18.8
Site %								
UBC	21.4	14.5	0.22	0.18	22.2	8.6	18.8
U of A	50.0	43.5			16.7	45.7	68.8
U of M	11.4	10.1			11.1	14.3	0.0
Queen’s	17.1	31.9			50.0	31.4	12.5

*Note: ^a^*p-*value: t-test for age and SES score between groups; non-parametric chi-square test for sex, handedness; Wilcoxon Rank Sum test for current caregiver, ethnicity, site. ^b^*n* = 132, handedness unknown for seven participants. ^c^Results reported for independent groups *t-*test with equal variances not assumed, results unchanged after a non-parametric Mann-Whitney U test given uneven ranges in distributions for the PAE and control groups (*p* = 0.07). *Note*: FAS, fetal alcohol syndrome; pFAS, partial fetal alcohol syndrome; ARND, alcohol-related neurodevelopmental disorder; PAE, prenatal alcohol exposure; SES, socioeconomic status; UBC, University of British Columbia; U of A, University of Alberta; U of M, University of Manitoba; Queen’s, Queen’s University*.

### SES and Cognitive Scores

Participants with PAE performed significantly worse than control children on all cognitive test scores, including measures of executive functioning, attention and working memory, memory, numerical ability, and word identification (all *p* < 0.001, all significant after correction for multiple comparisons, Table 2). We examined associations between SES and cognitive test scores within each group and found no significant associations.

**Table 2:** Cognitive test scores and correlations with SES in control and PAE groups.

	Controls		PAE^a^		Comparison	Controls (PAE vs. Controls)		PAE	
	*n*	*M* ± SD	*n*	*M* ± SD	*p* (*d*)	*r*	*p*	*r*	*p*
Executive Function									
Animal Sorting	69	9.7 ± 3.4	65	7.3 ± 3.0	<0.001* (−0.76)	0.25	0.04	−0.12	0.34
Inhibition-Naming	68	9.7 ± 3.2	64	7.0 ± 3.9	<0.001* (−0.77)	0.04	0.75	−0.03	0.83
Inhibition-Inhibition	68	10.2 ± 3.8	63	6.7 ± 3.2	<0.001* (−1.00)	0.12	0.32	0.11	0.41
Inhibition-Switching	68	10.8 ± 2.7	61	7.3 ± 3.5	<0.001* (−1.11)	0.17	0.17	0.03	0.82
Behavioral Regulation Index	68	46.9 ± 7.2	63	68.4 ± 12.1	<0.001* (2.18)	−0.23	0.06	0.002	0.99
Metacognitive Index	68	54.0 ± 14.0	63	65.4 ± 11.2	<0.001* (0.91)	−0.11	0.37	0.07	0.57
Attention and Working Memory									
Auditory Attention	69	11.1 ± 2.4	65	7.6 ± 4.0	<0.001* (−1.06)	0.02	0.87	0.02	0.89
Response Set	69	11.7 ± 2.6	65	10.0 ± 3.0	<0.001* (−0.63)	0.008	0.95	0.20	0.11
Digit Recall	64	100.5 ± 17.0	64	86.3 ± 13.4	<0.001* (−0.94)	0.13	0.31	0.15	0.23
Block Recall	63	103.0 ± 16.2	63	90.3 ± 16.0	<0.001* (−0.80)	0.14	0.29	0.25	0.05
Memory									
Memory for Names	69	9.9 ± 2.9	64	7.2 ± 3.3	<0.001* (−0.85)	−0.06	0.61	−0.19	0.14
Memory for Names Delayed	69	9.6 ± 2.5	64	7.3 ± 3.3	<0.001* (−0.81)	0.06	0.61	−0.21	0.10
Numerical Ability									
Quantitative Concepts	70	106.6 ± 11.6	68	82.7 ± 20.0	<0.001* (−1.48)	0.21	0.08	0.20	0.10
Word Identification	70	106.9 ± 11.5	68	90.5 ± 14.7	<0.001* (−1.26)	0.23	0.06	0.11	0.38

Note. ^a^Between-group comparisons for controls vs. PAE were conducted using between groups *t*-tests. Uncorrected *p*-values are shown. *Indicates a *p*-value met (Benjamini and Hochberg, 1995, 2000) false discovery rate criterion [(i/m)Q] where i = individual p-value rank, *m* = 14 (total number of tests), and *Q* = 0.10; PAE = prenatal alcohol exposure. SES = socioeconomic status.

### SES Correlated With Brain Volumes in Controls but not in PAE

All measured brain volumes were significantly smaller in children with PAE compared to controls after accounting for age and sex (Table 3). Most brain volumes for children with PAE were between 4 and 8% smaller compared to controls, with larger discrepancies for overall cerebellum WM and a deep gray matter structure (caudate; 10%, and 12% smaller, respectively). We examined relationships between SES and brain volumes within each of the PAE and control groups and found that SES was positively associated with subcortical structures including the hippocampus and amygdala for control children (Table 4, Figure 1). In contrast, no significant associations were observed between SES and brain volumes for children with PAE. Follow-up exploratory hierarchical linear regressions revealed no significant interactions between SES and group for any brain volumes, though diagnostic group (control vs. PAE) was significantly associated with 11 of 13 brain volumes (excluding cerebellum WM, *p* = 0.050, and amygdala, *p* = 0.051) in the overall sample (Supplementary Table S1).

**Table 3:** Regional brain volumes in control and PAE groups.

	Brain volume (*M* ± *SD*, cm^3^)		Comparison (PAE vs. Controls)^a^	
	Controls (*n* = 70)	PAE (*n* = 69)	Diff (%)^b^	*p* ($\eta_{\text{p}}^{2}$)
Total cerebrum^c^	1224 ± 122	1153 ± 121	−5.8	<0.001* (0.12)
Total GM^d^	751 ± 72	706 ± 73	−6.0	<0.001* (0.11)
Total subcortical GM^e^	51 ± 4.3	47 ± 4.8	−7.8	<0.001* (0.16)
Cortical GM	550 ± 59	521 ± 62	−5.3	0.002* (0.07)
Cerebrum WM	473 ± 59	447 ± 59	−5.5	<0.001* (0.09)
Cerebellum GM	120 ± 17	110 ± 13	−8.3	<0.001* (0.10)
Cerebellum WM	29.7 ± 8.4	26.8 ± 6.0	−9.8	0.030* (0.03)
Hippocampus	8.7 ± 0.9	8.1 ± 1.1	−6.9	0.001* (0.07)
Amygdala	3.1 ± 0.4	3.0 ± 0.4	−3.2	0.016* (0.04)
Thalamus	16.2 ± 1.5	15.2 ± 1.7	−6.2	<0.001* (0.14)
Caudate	7.8 ± 1.0	6.9 ± 1.1	−11.5	<0.001* (0.15)
Putamen	11.6 ± 1.3	11.0 ± 1.3	−5.2	0.002* (0.07)
Pallidum	3.5 ± 0.4	3.2 ± 0.5	−8.6	<0.001* (0.14)

Note. ^a^Comparisons were evaluated using analysis of covariance (ANCOVA), with age and sex as covariates. ^b^Difference % was calculated as (100 * [(volume in PAE) − (volume in controls)]/[(volume in controls)]. ^c^Total Cerebrum Volume = total gray matter (GM) + total white matter (WM). ^d^Total GM = subcortical GM + cortical GM. ^e^Total Subcortical Gray Matter = Hippocampus + Amygdala + Thalamus + Caudate + Putamen + Pallidum. *Indicates a *p*-value met (Benjamini and Hochberg, 1995, 2000) false discovery rate criterion [(i/m)Q] where i = individual p-value rank, *m* = 13 (total number of tests), and Q = 0.10.

**Table 4:** Associations between regional brain volumes and SES in control and PAE groups.

	Controls (*n* = 70)			PAE (*n* = 69)		
	*b*	SE	*p*	*b*	SE	*p*
Total Cerebrum^a^						
Age	−1.51	4.34	0.729	0.55	4.54	0.905
Sex	122.91	25.84	<0.001*	70.91	28.32	0.015
SES	2.99	1.41	0.038	0.52	1.01	0.605
Total GM^b^						
Age	−5.09	2.52	0.047	−4.92	2.73	0.076
Sex	67.75	15.01	<0.001*	36.69	17.02	0.035
SES	1.51	0.82	0.070	0.39	0.61	0.524
Total Subcortical GM^c^						
Age	0.09	0.16	0.581	0.13	0.19	0.493
Sex	3.88	0.95	<0.001*	2.04	1.16	0.082
SES	0.09	0.05	0.083	0.01	0.04	0.820
Cortical GM						
Age	−4.34	2.19	0.051	−4.79	2.32	0.043
Sex	48.36	13.04	<0.001*	29.01	14.46	0.049
SES	0.88	0.71	0.219	0.28	0.52	0.587
Cerebrum WM						
Age	3.59	2.14	0.098	5.46	2.10	0.012
Sex	55.16	12.73	<0.001*	34.22	13.10	0.011
SES	1.48	0.69	0.037	0.14	0.47	0.772
Cerebellum GM						
Age	−1.07	0.64	0.098	−0.51	0.49	0.306
Sex	13.18	3.80	0.001^*	4.80	3.06	0.122
SES	0.43	0.21	0.041	0.10	0.11	0.374
Cerebellum WM						
Age	−0.18	0.35	0.611	−0.09	0.24	0.697
Sex	−1.34	2.10	0.527	0.50	1.48	0.739
SES	0.12	0.12	0.306	−0.01	0.05	0.931
Hippocampus						
Age	0.02	0.04	0.516	0.02	0.04	0.694
Sex	0.68	0.21	0.002^*	0.18	0.27	0.507
SES	0.03	0.01	0.009^*	0.004	0.01	0.677
Amygdala						
Age	0.03	0.01	0.069	0.004	0.02	0.808
Sex	0.26	0.08	0.003^*	0.16	0.11	0.140
SES	0.01	0.01	0.005^*	0.003	0.004	0.408
Thalamus						
Age	0.10	0.05	0.076	0.10	0.06	0.130
Sex	1.71	0.32	<0.001*	0.66	0.39	0.097
SES	−0.002	0.02	0.917	−0.01	0.01	0.458
Caudate						
Age	0.02	0.04	0.694	0.02	0.04	0.641
Sex	0.38	0.24	0.123	−0.01	0.27	0.983
SES	0.02	0.01	0.259	−0.001	0.01	0.955
Putamen						
Age	−0.07	0.05	0.174	−0.02	0.05	0.744
Sex	0.64	0.32	0.050	0.84	0.31	0.009^*
SES	0.03	0.02	0.112	0.01	0.01	0.386
Pallidum						
Age	0.001	0.02	0.947	0.01	0.02	0.731
Sex	0.23	0.09	0.018^*	0.21	0.11	0.060
SES	0.01	0.01	0.252	0.004	0.004	0.367

Note. Unstandardized regression coefficients shown for age, sex, and SES, for brain volumes, separately for PAE and controls. ^a^Total Cerebrum Volume = total gray matter (GM) + total white matter (WM). ^b^Total GM = subcortical GM + cortical GM. ^c^Total Subcortical Gray Matter = Hippocampus + Amygdala + Thalamus + Caudate + Putamen + Pallidum. *Indicates a p-value met (Benjamini and Hochberg, 1995, 2000) false discovery rate criterion [(i/m)Q] where i = individual p-value rank, *m* = 39 (total number of tests), and *Q* = 0.10. SES = socioeconomic status. PAE = prenatal alcohol exposure.

## Discussion

### SES and Brain Volumes

Complementing a range of prior studies, our findings indicated significantly smaller brain volumes across cerebrum, cerebellum, and deep gray matter volumes in children with PAE compared to neurotypically developing children (Norman et al., 2009; Lebel et al., 2011; Nardelli et al., 2011; Uban et al., 2020). Subgroup analyses showed associations between higher SES and larger volumes of specific subcortical structures including the hippocampus and amygdala in neurotypically developing children, but not in the PAE cohort; both covering the same age range of 7–18 years. Though we did not see differential patterns of association for SES by group, our findings complement results recently reported by Uban et al. (2020) in an independent cohort of similarly-aged children with PAE and controls. They found differential patterns of association for SES indicators and regional brain volumes in children with PAE vs. controls (hippocampus, nucleus accumbens, and ventral diencephalon), as well as within-group associations between SES indicators and multiple regional volumes including the amygdala in only controls, but not children with PAE.

Our results are also consistent with previous studies indicating that SES is positively correlated with brain volumes in typically developing children and adolescents (Hackman and Farah, 2009; Noble et al., 2012; Jednoróg et al., 2012; Ellwood-Lowe et al., 2018; Yu et al., 2018; McDermott et al., 2019). This includes a recent large scale longitudinal study of children ages 5–25 years (*N* = 623) where SES was positively associated with total brain volume, both gray and WM volume, cortical volume, in addition to subcortical structures including both the hippocampus and amygdala in a cross-sectional examination of the full sample (McDermott et al., 2019). These associations also remained stable in a subset of 344 individuals with multiple scans suggesting that intra-individual age-related change did not modify the SES-volume relationship. Our results also fit with other cross-sectional studies. For instance, lower SES has been associated with lower cortical gray matter, hippocampal, and amygdala volumes in typically developing 3–15-year-olds (Luby et al., 2013); regional volumes of the hippocampus and amygdala in 5–17-year-olds (Noble et al., 2012); regional volumes of the middle temporal gyri, left fusiform, and right inferior occipito-temporal gyri in typically developing 8–10-year-olds (Jednoróg et al., 2012); and hippocampal volume in 10–24-year-old girls (Ellwood-Lowe et al., 2018) and children aged 8–12 years (Yu et al., 2018). The negative effect on regional brain morphology in individuals from lower SES families may relate to the lack of nutrients during prenatal or postpartum development (Ivanovic et al., 2002; Pechey and Monsivais, 2015; Ranjit et al., 2015), decreased overall health (Adler and Ostrove, 2006) or raised stress levels (Bradley and Corwyn, 2002; Hackman and Farah, 2009; Hackman et al., 2010; Pagliaccio et al., 2014), all of which could have an impact on the developing brain.

The influence of SES on brain structure seems to be a long-term effect where early years spent in a lower SES environment can affect the brain into and beyond adulthood, such as one previous study which reported that the hippocampus volume at 64 years of age was positively correlated with familial SES status at 11 years (Staff et al., 2012). Another study showed that SES in childhood at age 11 years positively correlated with cerebellum volumes (lower SES predicted less volume) at 35–64 years (Cavanagh et al., 2013). As noted above, McDermott et al. (2019) demonstrated stable associations between SES and multiple indicators of brain volume in a longitudinal model for children and young adults aged 5–25 years. However, there are exceptions, such as one study where childhood poverty was not found to be associated with brain volumes at 44–48 years, but interestingly bilateral hippocampal and amygdala volumes did correlate with the participants’ current financial status (Butterworth et al., 2012). Similarly, two studies did not find correlations between current SES and brain volumes in healthy participants of ~30 years, but did show links between lower SES and reduced gray matter volumes in individuals with schizophrenia over this age range (Takayanagi et al., 2010; Yeo et al., 2014).

Although brain volume often forms the primary focus in imaging research, SES studies have also examined cortical thickness and diffusion tensor imaging (DTI) of WM. For instance, in a study of neurotypical children ages 4–18 years, the thicker cortex in the left superior frontal gyrus and right anterior cingulate gyrus was found to correlate with higher parental education (Lawson et al., 2013). Other typical development studies have addressed SES and WM volume and DTI metrics (for a review see Brito and Noble, 2014), with greater SES, linked to larger overall WM volumes over ages 3–15 years (Luby et al., 2013), and higher education to greater fractional anisotropy in the superior longitudinal fasciculus and cingulum bundle over 17–23 years (Noble et al., 2013).

In contrast to the controls, SES did not correlate with brain volumes for children with PAE in the current study. As indicated, this finding corresponds with results reported by Uban et al. (2020) in their recent U.S. cohort of similarly aged children, lending further independent support to our results. Several possible explanations may account for this outcome. The lack of association could suggest that central nervous system structures were already altered by alcohol exposure before birth and that subsequent SES conditions did not mediate brain volume. For instance, in a cohort of 28 children with PAE and 56 controls, ages 6–17 years, Nardelli et al. (2011) found the largest relative reductions in total deep gray matter volumes for children with PAE vs. controls from a range of imaging volumes, raising the possibility that the attenuation of deep gray matter brain volume in children with PAE could be overwhelming any potential SES effects, as observed in specific subcortical structures for neurotypically developing children in the current study. Uban et al. (2020) similarly suggest that reduced neuroplasticity following PAE, in addition to increased susceptibility to stress, may result in attenuation of brain-SES associations frequently observed in neurotypically developing children. PAE has many potential mechanisms of injury on fetal brain development, including disruption and impairment of cellular energetics, glucose utilization and transport, the timing of cell acquisition/dysregulation, gene expression, protein, and DNA synthesis, cell to cell interactions, growth factor signaling, or cell death, in addition to others (for reviews, see Goodlett et al., 2005; Young et al., 2014). A possible lack of association of brain volumes with SES could also be expected in the PAE population given that structural brain alterations are already seen in early neonates with PAE (Taylor et al., 2015; Donald et al., 2016). These prenatal PAE injuries could then extend throughout neurodevelopment in children and adolescents, as shown by MRI measures of cortical thickness and WM (Zhou et al., 2011; Treit et al., 2014).

### SES and Cognitive Scores

Consistent with established literature, our results indicated that children with PAE performed substantially worse than neurotypically developing children across all areas of cognitive functioning assessed (Mattson et al., 2019; Uban et al., 2020). After accounting for multiple comparisons we did not find positive associations between SES and cognitive abilities including measures of executive functioning, attention and working memory, memory, mathematical/numerical ability, and word reading, for either typically developing children or children with PAE, though trends were apparent for indicators of executive functioning and math in controls. Among controls, this finding stands in contrast to an array of studies that generally demonstrate positive associations between SES and a variety of cognitive indicators (for reviews see Bradley and Corwyn, 2002; Hackman and Farah, 2009; Hackman et al., 2010; Farah, 2017). Examples include overall intellectual functioning (von Stumm and Plomin, 2015) executive functions (Ardila et al., 2005; Last et al., 2018), working memory (Engel et al., 2008; Evans and Schamberg, 2009; Evans and Fuller-Rowell, 2013), and mathematical achievement (Kobrosly et al., 2011), all being associated with SES in developmental studies. Our current findings may be the result of sampling, with our neurotypically developing subgroup being best characterized as a healthy group of children drawn largely from moderate to high socioeconomic backgrounds. As well, early vs. later childhood or adulthood socioeconomic disadvantage is more strongly associated with later cognitive achievement in neurotypically developing children, and this remains understudied in FASD (e.g., Duncan et al., 1998). As such, these findings should be interpreted in the context of future studies that take into consideration the need to assess more detailed information on child environment (e.g., adversities, other exposures, placement disruptions and care stability including shifts in SES) to best understand the impact of SES on cognitive development in children, particularly for those with PAE.

### Limitations

In the current study, several limitations render it premature to determine whether the absence of associations between current SES and brain volume can be conclusively interpreted. The suboptimal number of children with PAE from very low SES caregiving circumstances, coupled with fewer control children from low SES backgrounds, limits our ability to draw firm conclusions and suggests the need for further research in this vulnerable population. It warrants highlighting that there are practical challenges involved in enrolling children and families of low SES and complex disabilities into voluntary studies involving multiple visits and MRI scans. Future research should include appropriate recruitment and support strategies to ensure that research participation is accessible for a broad range of families.

The cross-sectional study also limits the ability to draw longitudinal and/or developmental conclusions about the degree of intra-individual change that could be accounted for as a result of environmental exposures or changes in SES over time. As well, we did not capture information about other relevant risk factors, including additional adverse environmental experiences or stressors, or other prenatal exposures such as drugs or cigarettes, nor could we control for additional potential covariates, such as ethnicity. As such, the current findings are best framed as exploratory and can be used to direct future methodological design and approaches to explore the impact of SES during sensitive developmental years for children with PAE.

Another important consideration centers on the nature of caregiving placement differences between our typically developing and PAE groups. Specifically, there is a greater likelihood that our assessment of current SES better reflects a stable environmental marker across development in controls given that most resided with their biological parents. This stands in contrast to the PAE group, where approximately 30% of children were living in foster care or another guardianship arrangement, with the majority (60%) living in adoptive families. This is common in PAE samples, and consistent with the Uban et al. (2020) cohort, where 74% of children with PAE were living in adoptive families compared to only 8% of control children. In our study, we did not have data concerning the stability or length of caregiving placements for participants in either group, serving as a further potential confound in any association between SES and brain volumes for children with PAE. However, Uban et al. (2020) were able to control for the length of placement specifically among adoptive families and found that most group-by-SES interactions remained significant across subcortical brain volumes. That said, there remains a clear need to consider the potential differential impact of SES on biological vs. adoptive families and other caregiving configurations in future studies.

The assessment of current vs. prenatal or early perinatal SES also warrants consideration. Several studies have assessed current SES using a variety of approaches (e.g., parent income in the last year, parental education, etc.), including the Uban et al. (2020) PAE cohort, as well as in older neurotypically developing children and adolescents, with several showing SES-brain volume associations (for a review see Brito and Noble, 2014). Cavanagh et al. (2013) also studied adults aged 36–65 years and found that both early life SES (e.g., childhood poverty, paternal social class, etc.) and current SES predicted cerebellar gray matter volume, with current SES adding significantly to predictive models that already considered early-life SES, further speaking to the importance of both current SES and early life environmental impacts. These findings suggest that our assessment of current SES is consistent with approaches commonly adopted in the relevant and rapidly unfolding literature, though, there remains a research gap comparing the relative impact of SES in early vs. later developmental periods, or from a longitudinal approach, using brain imaging.

Also, in the current study, although children with PAE who were living in higher SES families may have been provided better opportunity to access the essential nutrients, interventions, and broad environmental conditions for positive development (Ivanovic et al., 2002; Pechey and Monsivais, 2015; Ranjit et al., 2015), this may not have translated into associations with brain volume in these children for the overall sample. As such, the lack of association between SES with brain volumes in children with PAE could be explained by a variety of factors, including the substantial effect of PAE, the variability of time spent, number, and quality of caregiving placements, and other factors not measured in the current study (e.g., maternal education, variability in the level and timing of alcohol exposure during pregnancy, et cetera).

### Conclusion

This study observed that a higher current SES caregiving environment was associated with larger hippocampus and amygdala volumes in neurotypically developing children and adolescents, but not in those with PAE. Findings are consistent with an established SES literature for neurotypically developing children, and a single recent study of children with PAE. Several explanations warrant consideration, including the possibility that lack of brain volume-SES associations in children with PAE could result if the initial PAE-related injury overwhelms postpartum brain development such that later SES does not play as substantial a role. However, additional research accounting for a more comprehensive assessment of developmental trajectories for SES, as well as other pre and postnatal experiences and exposures in PAE cohorts that span the full SES spectrum, is required to inform firm conclusions. The current findings contribute to a growing body of evidence characterizing the impact of PAE on structural and cognitive brain outcomes in the context of additional pre and postnatal adversities, including SES.

## Data Availability Statement

The datasets generated for this study are available on request to the corresponding author.

## Ethics Statement

The studies involving human participants were reviewed and approved by the Human Research Ethics Boards at Queen’s University, the University of Alberta, the Children’s Hospital of Eastern Ontario, the University of Manitoba, and the University of British Columbia. Written informed consent to participate in this study was provided by the participants’ legal guardian.

## Author Contributions

KM, DZ, GL, and CB: completed the literature review, performed statistical analyses, data analysis/interpretation, and manuscript drafting and editing. DZ and GL: performed image analysis and quality assessment. All authors: study concepts/study design, data acquisition, manuscript review and revision, editing, and approval of the final manuscript.

## Conflict of Interest

The authors declare that the research was conducted in the absence of any commercial or financial relationships that could be construed as a potential conflict of interest.

## Footnotes

^1^A revised set of Canadian Diagnostic Guidelines for FASD were released subsequent to data collection, resulting in changes to diagnostic terminology and criteria.

^2^http://cbrain.mcgill.ca/
