# Supplementary material for: Current Socioeconomic Status Correlates With Brain Volumes in Healthy Children and Adolescents but Not in Children With Prenatal Alcohol Exposure
Source: Front Hum Neurosci. 2020 Jun 30;14:223. doi: 10.3389/fnhum.2020.00223 (PMC7344164; doi:10.3389/fnhum.2020.00223)
Supplement: Supplementary file 1 [file Table_1.pdf]

Supplementary Table 1. Associations between regional brain volume and SES-diagnosis interactions

	Age			Sex			Diagnosis			SES[d]			Diagnosis*SES[d]		
	b	SE	*p*	*b*	SE	*p*	*b*	SE	*p*	*b*	SE	*p*	*b*	SE	*p*
Total Cerebrum[a]	-.77	3.13	.806	98.96	19.11	<.001	74.23	19.42	<.001	1.85	2.42	.446	-2.38	1.77	.181
Total GM[b]	-5.21	1.85	.006	51.97	11.31	<.001	43.18	11.49	<.001	.71	1.43	.622	-1.09	1.05	.301
Total Subcortical GM[c]	.10	.12	.429	2.95	.75	<.001	3.62	.76	<.001	.07	.09	.460	-.08	.07	.254
Cortical GM	-4.72	1.59	.003	38.38	9.67	<.001	27.83	9.82	.005	.32	1.23	.793	-.59	.90	.510
Cerebrum WM	4.44	1.49	.003	44.99	9.09	<.001	31.06	9.24	.001	1.14	1.15	.323	-1.29	.84	.128
Cerebellum GM	-.83	.40	.041	9.06	2.44	<.001	9.19	2.48	<.001	.22	.31	.485	-.32	.23	.162
Cerebellum WM	-.12	.21	.561	-.38	1.27	.765	2.55	1.29	.050	.13	.16	.434	-.12	.12	.298
Hippocampus	.02	.03	.558	.42	.17	.015	.50	.17	.005	.02	.02	.302	-.03	.02	.100
Amygdala	.01	.01	.218	.20	.07	.003	.13	.07	.051	.01	.01	.377	-.01	.01	.097
Thalamus	.09	.04	.034	1.17	.25	<.001	1.22	.26	<.001	.02	.03	.568	-.01	.02	.745
Caudate	.02	.03	.597	.19	.18	.310	.84	.18	<.001	.02	.02	.492	-.02	.02	.368
Putamen	-.04	.04	.252	.75	.22	.001	.62	.23	.007	.01	.03	.804	-.02	.02	.398
Pallidum	.004	.01	.760	.22	.07	.003	.31	.07	<.001	-.001	.01	.886	-.002	.01	.730

Note. Unstandardized regression coefficients for final step of hierarchical models for each regional brain volume are shown. [a] Total Cerebrum Volume = total gray matter (GM) + total white matter (WM); [b] Total GM = subcortical GM + cortical GM. [c] Total Subcortical Gray Matter = Hippocampus + Amygdala + Thalamus + Caudate + Putamen + Pallidum. [d] = Mean centred SES. Values are not corrected for multiple comparisons given exploratory analyses. SES = socioeconomic status. PAE = prenatal alcohol exposure.
